# Calcium Citrate Amount and Gelatine Source Impact on Hydroxyapatite Formation in Bone Regeneration Material in Simulated Body Fluid

**DOI:** 10.3390/molecules29163925

**Published:** 2024-08-20

**Authors:** Yuejun Wang, Taishi Yokoi, Masaya Shimabukuro, Masakazu Kawashita

**Affiliations:** 1 Graduate School of Medical and Dental Sciences, Tokyo Medical and Dental University, 1-5-45 Yushima, Bunkyo-ku, Tokyo 113-8510, Japan; 2 Institute of Biomaterials and Bioengineering, Tokyo Medical and Dental University, 2-3-10 Kanda-Surugadai, Chiyoda-ku, Tokyo 101-0062, Japan; shimabukuro.bcr@tmd.ac.jp (M.S.); kawashita.bcr@tmd.ac.jp (M.K.)

**Keywords:** calcium citrate, fish gelatine, octacalcium phosphate, simulated body fluid test, hydroxyapatite

## Abstract

Bone grafting is crucial for bone regeneration. Recent studies have proposed the use of calcium citrate (CC) as a potential graft material. Notably, citrate does not inhibit hydroxyapatite (HAp) formation at specific calcium-to-citrate molar ratios. Octacalcium phosphate (OCP)/gelatine (Gel) composites, which are commonly produced from porcine Gel, are valued for their biodegradability and bone replacement capability. This study introduces fish Gel as an alternative to porcine Gel because of its wide acceptance and eco-friendliness. This is the first study to examine the interaction effects between two osteogenic materials, OCP/CC, and the influence of different gelatine matrix components on HAp formation in an SBF. Samples with varying CC contents were immersed in an SBF for 7 d and analysed using various techniques, confirming that high CC doses prevent HAp formation, whereas lower doses facilitate it. Notably, small-sized OCP/CC/porcine Gel composites exhibit a high HAp generation rate. Porcine Gel composites form denser HAp clusters, whereas fish Gel composites form larger spherical HAps. This suggests that lower CC doses not only avoid inhibiting HAp formation but also enhance it with the OCP/Gel composite. Compared with porcine Gel, fish Gel composites show less nucleation but an increased crystal growth for HAp.

## 1. Introduction

The reconstruction of large bone defects caused by trauma, tumours, or infection is a key problem in reconstructive surgery. Bone tissue possesses the capacity for self-repair and regeneration, allowing minor defects to heal on their own without additional treatment [1]. However, if the bone defect is approximately the critical size threshold (more than 2 cm) or results in a loss of more than 50% of the bone circumference [2], conditions such as non-union, malunion, or pathological fractures can occur [3]. Autologous bone grafts are the gold standard for bone regeneration. However, the supply of autologous bone is limited, and the removal of autologous bone can lead to infection and pain at the donor site. Bone allografts are a secondary option for orthopaedic treatment because they are readily available in various forms and in large quantities. Nevertheless, reduced osteoinductivity may result in healing that is not as effective as that achieved using autologous grafts. Consequently, there is an increasing demand for artificial bone materials, such as calcium carbonate and calcium phosphate (CaP), for the regeneration of bone tissue [4].

Octacalcium phosphate (OCP) is a form of calcium phosphate that acts as a precursor to hydroxyapatite (HAp) and shows superior bone regeneration capabilities. Imaizumi et al. reported that OCP promotes faster bone growth than HAp materials do [5]. In addition, it surpasses the biodegradation rate of β-tricalcium phosphate while transforming into calcium-deficient HAp [6]. Gelatine (Gel) is a derivative of denatured collagen [7]. OCP is valued for its biodegradability and biocompatibility and serves as an economical hydrogel [8]. The combination of OCP and Gel yields a composite that not only offers enhanced handling characteristics and facilitates easier clinical use but also augments osteoconductivity [9]. This combination accelerates biodegradation and is closely followed by new bone formation, making it a highly effective option for bone repair and regeneration in medical treatments [10]. Existing research on OCP/Gel composites has primarily focused on porcine Gel, overlooking the potential benefits of combining OCP with other Gels. The main sources of Gel are pigs, bovines, and fish. Fish Gel is a valuable alternative to porcine Gel, thereby broadening the spectrum of choices in biomaterial research. Fish Gel is recognised as an eco-friendly option that utilises byproducts of the fish-processing industry because it is compatible across various cultures and religions and free from concerns related to bovine spongiform encephalopathy [11]. This not only contributes to environmental sustainability but also underscores the importance of resource efficiency [12]. While fish and porcine Gels differ in their protein content and amino acid composition [13], their distinct properties emphasise their potential for various applications in promoting bone regeneration. By combining fish Gel with an OCP/Gel composite, the versatility and applicability of biomaterials can be enhanced to meet a broader range of scientific and practical needs.

Considering the benefits of combining OCP with Gel for bone regeneration, studying other materials that can synergistically enhance these effects would be valuable. Calcium citrate (CC), a calcium salt of citric acid, is a promising material for bone repair. Previous studies have indicated that concentrations of 2–4 mM Ca^2+^ are conducive to osteoblast proliferation and survival, whereas slightly higher concentrations of 6–8 mM are preferable for osteoblast differentiation and matrix mineralisation in both two- and three-dimensional cultures. However, Ca^2+^ concentrations exceeding 10 mM are cytotoxic [14]. In a simulated body fluid (SBF), the Ca^2+^ amount released from CC is approximately 7 mmol/L [15], which adequately influences osteoblast differentiation and proliferation in vivo, rendering CC a beneficial supplement for patients with osteoporosis. Moreover, the degradation of CC does not significantly alter the pH of body fluids, maintaining a pH range of 7.20–7.46. This minimal fluctuation in pH suggests low irritability to the human body and underscores the excellent biocompatibility of CC, making it a viable choice for artificial bone-repair materials. Citrate ions play a crucial role in bone regeneration. They are integral to the apatite nanocrystal/collagen complex and enhance bone formation and mineralisation [16]. Exogenous citrate supplementation enhances the expression of genes related to bone development, such as alkaline phosphatase and osterix; fosters the maturation of the osteoblast phenotype; and increases the osteoinductivity of implants [17]. However, citrate alone does not inhibit HAp formation within a specific range of calcium-to-citrate molar ratios [18].

Considering the promising results of OCP/Gel composites and the potential benefits of combining them with additional materials, this study aimed to elucidate the circumstances under which CC synergistically interacted with OCP to optimise bone regeneration outcomes. Previous studies have only explained the effect of citric acid on the nucleation of HAp in an SBF. However, when citric acid is converted to a potential osteogenic material CC and combined with OCP, it is crucial to understand the mechanisms through which OCP and CC interact and determine which of these influences has a greater effect on HAp nucleation. Therefore, this study comprehensively explored their combined effects as well as the corresponding underlying mechanisms. Additionally, the impact of incorporating different types of gelatine on this interaction was investigated.

The focus will be primarily on the characterisation of HAp formation within OCP/CC/Gel systems in an SBF, with the aim of providing deeper insights into the mechanical aspects underlying effective bone tissue engineering.

## 2. Results

### 2.1. Conversion of Different Amounts of Calcium Citrate Powder Immersed in Simulated Body Fluid

**Figure 1:**
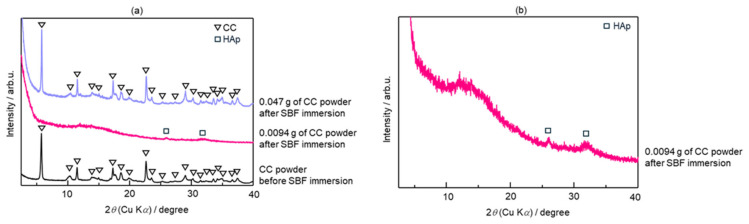
(**a**) X-ray diffraction (XRD) patterns of calcium citrate (CC) powder in different amounts before and after immersion in a simulated body fluid (SBF). (**b**) Enlarged XRD pattern of 0.0094 g of CC powder after immersion in SBF.

Figure 1a shows the X-ray diffraction (XRD) patterns of the CC powder before immersion in an SBF and 0.0094 and 0.047 g of the CC powder soaked in an SBF. After immersion in the SBF, the CC sample with a mass of 0.0094 g demonstrated HAp characteristic peaks at 2*θ* positions of 26° and 32° (Figure 1b), signifying its complete conversion to HAp. Conversely, the XRD spectrum of the sample with 0.047 g of CC remained largely unchanged, closely resembling that of the initial CC powder before SBF soaking. This result indicated that a smaller quantity of CC (0.0094 g) underwent a full transformation into HAp upon SBF immersion, whereas a larger quantity (0.047 g) retained its original crystalline phase, exhibiting no significant conversion.

**Figure 2:**
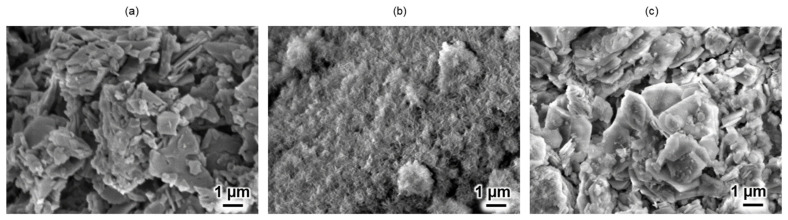
Scanning electron microscopy (SEM) images of CC powder under different conditions. (**a**) Unsoaked CC, showing its original surface morphology and particle distribution, and (**b**) 0.0094 and (**c**) 0.047 g of CC powder soaked in SBF.

The crystal morphologies of unsoaked CC (Figure 2a) and 0.0094 and 0.047 g of CC soaked in an SBF (Figure 2b and Figure 2c, respectively) were observed using scanning electron microscopy (SEM). The unsoaked CC powder exhibits a small, fragmented morphology, as shown in Figure 2a. After immersion in the SBF, the morphology of the sample containing 0.0094 g of CC powder transformed into rod-like clusters (Figure 2b).

In contrast, the sample containing 0.047 g of CC powder retained the initial small fragmentary morphology of CC, as shown in Figure 2c.

**Figure 3:**
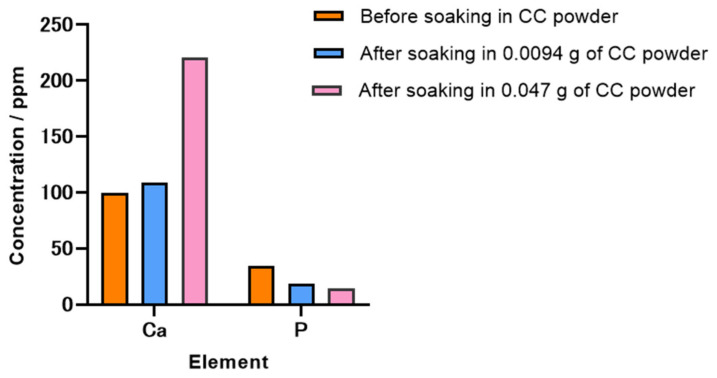
Variations in calcium and phosphate ion concentrations in SBF before and after immersion of 0.0094 and 0.047 g of CC powder. Changes in ion concentration indicate interaction between different amounts of CC and SBF.

Figure 3 illustrates the variations in calcium and phosphate ion concentrations in the SBF before and after the immersion of 0.0094 and 0.047 g of CC powder, evaluated using inductively coupled plasma optical emission spectroscopy (ICP-OES).

After immersion in 0.0094 g of CC powder, a marginal increase in the calcium concentration and a reduction in the phosphate concentration were observed in the SBF. These results implied that most of the calcium ions released from CC were probably used in the synthesis of HAp, while the phosphate ions were concurrently consumed in the SBF. However, immersion in 0.047 g of CC powder resulted in a notable increase in the calcium levels, with a concurrent decrease in the phosphate levels. The pronounced elevation in the calcium concentration indicated that the calcium ions released from CC likely did not participate in the synthesis of HAp.

**Figure 4:**
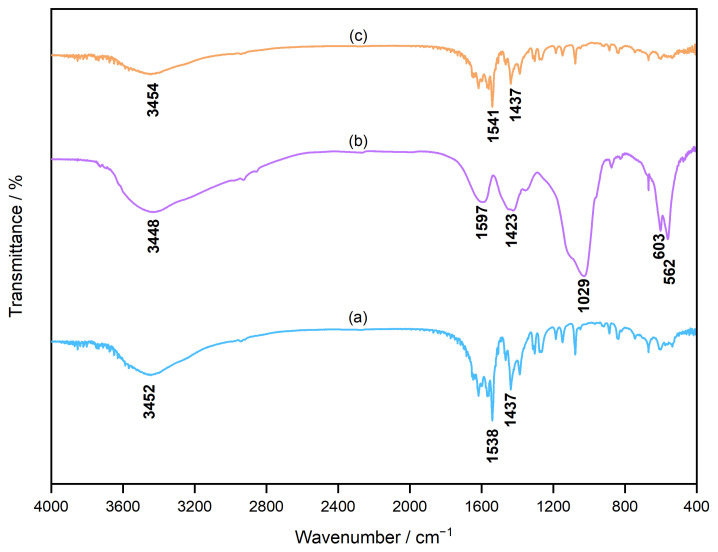
Fourier-transform infrared (FTIR) spectra of CC powder under different conditions: (a) CC before being soaked in SBF, showing baseline spectral characteristics of the material. (b) An amount of 0.0094 g of CC after being soaked in SBF, and (c) 0.047 g of CC powder after being soaked in SBF.

Fourier-transform infrared (FTIR) spectroscopy was employed to characterise the different functional groups of the samples with 0.0094 and 0.047 g of CC powder before and after soaking in the SBF. The FTIR spectra of the unsoaked and soaked samples in the range of 4000–400 cm^−1^ are shown in Figure 4. The spectra of (a) the unsoaked CC samples and (c) 0.047 g of CC powder soaked in the SBF (Figure 4) present broad bands at 3452 and 3545 cm^−1^ that are attributed to the O–H bonds in crystal water or other moieties. In CC, because the carboxy group could be coordinated to the calcium ion that formed carboxylate, strong characteristic absorption peaks were detected at (a) 1538, 1437, and (c) 1541, 1437 cm^−1^. Moreover, the characteristic FTIR absorption bands of the three carboxylate ions in CC were split into two peaks owing to the strong coupling effect of the antisymmetric stretching vibration at (a) 1538 and (c) 1541 cm^−1^, whose infrared activity was significantly high, and the symmetric stretching vibration at 1437 cm^−1^, which had a relatively low infrared activity [19]. A peak was observed at 3448 cm^−1^ in the spectrum of the sample containing 0.0094 g of CC powder after soaking in the SBF, which was attributed to the adsorbed moiety of OH^−^. The presence of the PO_4_^3−^ group was confirmed by the observation of peaks at 562, 603, and 1029 cm^−1^. In addition, peaks for CO_3_^2−^ were observed at 1423 and 1597 cm^−1^, which were characteristic of HAp [20]. The FTIR results also confirmed that only a small amount (0.0094 g) of CC was converted into HAp after the SBF test, whereas a larger dosage (0.047 g) of CC remained unconverted and retained its original structure.

### 2.2. Conversion of Octacalcium Phosphate/Calcium Citrate/Porcine Gelatine Composite Samples Produced in 96- and 24-Well Plates Immersed in Simulated Body Fluid

**Figure 5:**
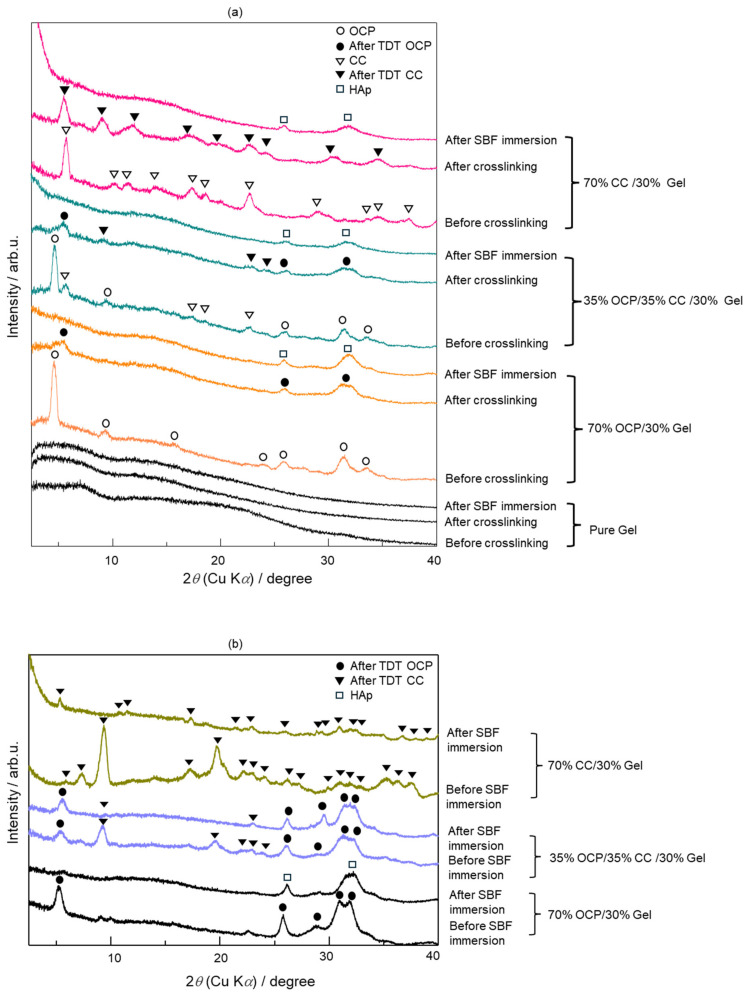
XRD patterns of octacalcium phosphate (OCP)/CC/gelatine (Gel) composite samples under different conditions: (**a**) before and after crosslinking and SBF testing prepared in 96-well plates, (**b**) before and after SBF testing prepared in 24-well plates. The labels “After TDT OCP” and “After TDT CC” refer to the new peaks observed in the crystalline phase of the OCP/CC/Gel samples, after undergoing thermal dehydration treatment (TDT) during the crossing process. These labels indicate the crystalline phase changes induced by the TDT.

Figure 5 shows the XRD patterns of the OCP/CC/Gel composite samples synthesised in 96- and 24-well plates (Figure 5a and Figure 5b, respectively) before and after immersion in an SBF for 7 d. Figure 5a shows the XRD patterns of the samples before and after crosslinking. After undergoing the thermal dehydration treatment (TDT) during crosslinking, changes in the crystalline phase peaks were observed in the OCP/CC/Gel samples, which are labeled as “After TDT OCP” and “After TDT CC”. These labels indicate the crystalline phase changes induced by TDT. The changes in the XRD patterns demonstrated that the 70% OCP/30% Gel, 35% OCP/35% CC/30% Gel, and 70% CC/30% Gel composites synthesised in a 96-well plate tended to convert to an apatite structure after being immersed in an SBF because they all contained a characteristic peak at the 2*θ* position of 32°, which agreed with the expected results from the apatite structure shown in Figure 5a.

Figure 5b shows the XRD patterns of the 70% OCP/30% Gel, 35% OCP/35% CC/30% Gel, and 70% CC/30% Gel composites synthesised using a 24-well plate. After immersion in an SBF, only the 70% OCP/30% Gel sample formed HAp. The samples that contained CC, namely the 35% OCP/35% CC/30% Gel and 70% CC/30% Gel composites, did not form HAp. This suggested that the inclusion of CC in the samples prepared in 24-well plates inhibited HAp formation in an SBF.

**Figure 6:**
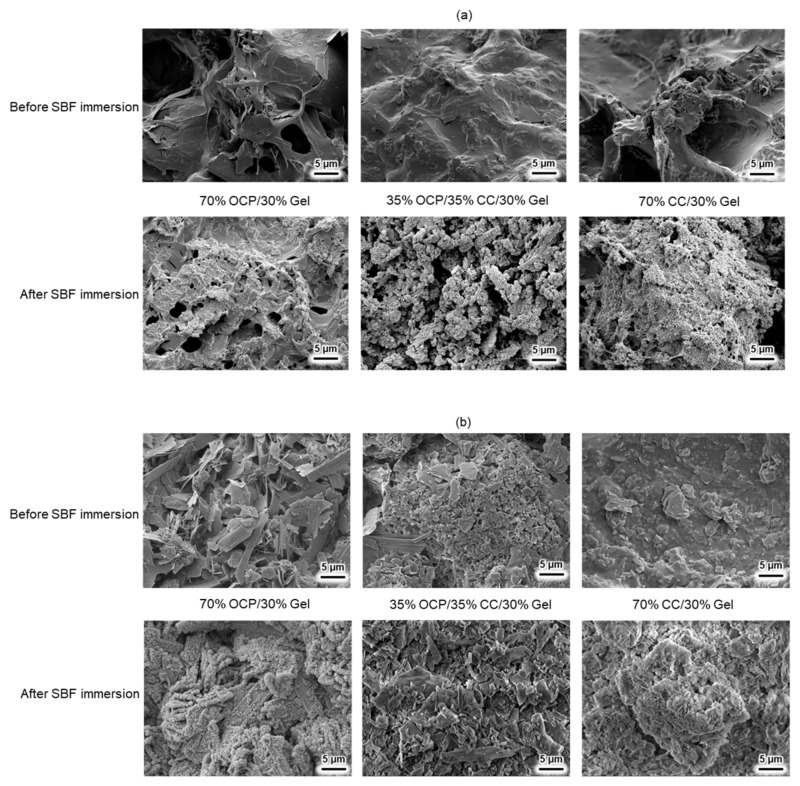
SEM images of OCP/CC/Gel composite samples prepared in (**a**) 96- and (**b**) 24-well plates before and after SBF testing.

Figure 6 shows the SEM images of the 70% OCP/30% Gel, 35% OCP/35% CC/30% Gel, and 70% CC/30% Gel composites, which were prepared using 96- and 24-well plates, before and after immersion in an SBF. Initially, the 70% OCP/30% Gel samples exhibited a morphology characterised by blade-shaped OCP structures interspersed within the Gel matrix. The 70% CC/30% Gel samples displayed a morphology comprising small, fragmented pieces of CC distributed throughout the Gel matrix. The 35% OCP/35% CC/30% Gel samples exhibited a mixed morphology, with both fragmented CC and blade-shaped OCP structures present within the gel matrix.

After immersion in the SBF, the materials prepared in the 96-well plates underwent a complete transformation into HAp structures, as confirmed by the XRD patterns (Figure 5a), which showed that the structure transformed into a cluster-like HAp structure, as shown in Figure 6a. Notably, the sample with 35% OCP/35% CC/30% Gel exhibited the highest material surface generation rate of HAp formation, indicating a synergistic effect between CC and OCP in promoting HAp formation in the SBF. This indicated that the 35% OCP/35% CC/30% Gel composite was a promising material for bone regeneration applications.

Conversely, as shown in Figure 6b, the materials synthesised in 24-well plates exhibited different behaviours. After immersion in the SBF, only the surfaces of the 70% OCP/30% Gel samples exhibited HAp structures. The samples containing CC, including the 35% OCP/35% CC/30% Gel and pure 70% CC/30% Gel composites, did not undergo conversion to HAp. Furthermore, owing to the high biodegradability of the Gel component, dissolution occurred within 7 d, resulting in the final samples retaining only their ceramic constituents. As a result, the SEM images of samples synthesised in 24-well plates after the SBF test revealed the exclusive presence of OCP and CC structures, and the Gel matrix was no longer observable.

### 2.3. Conversion of Octacalcium Phosphate/Calcium Citrate/Gelatine Composite Samples with Different Gelatine Sources Immersed in Simulated Body Fluid

The experimental results indicated that the samples prepared in a 96-well plate could form HAp after immersion in the SBF. To further explore the influence of the Gel source on the nucleation and growth of HAp, the samples prepared with porcine and fish Gels in 96-well plates were compared.

**Figure 7:**
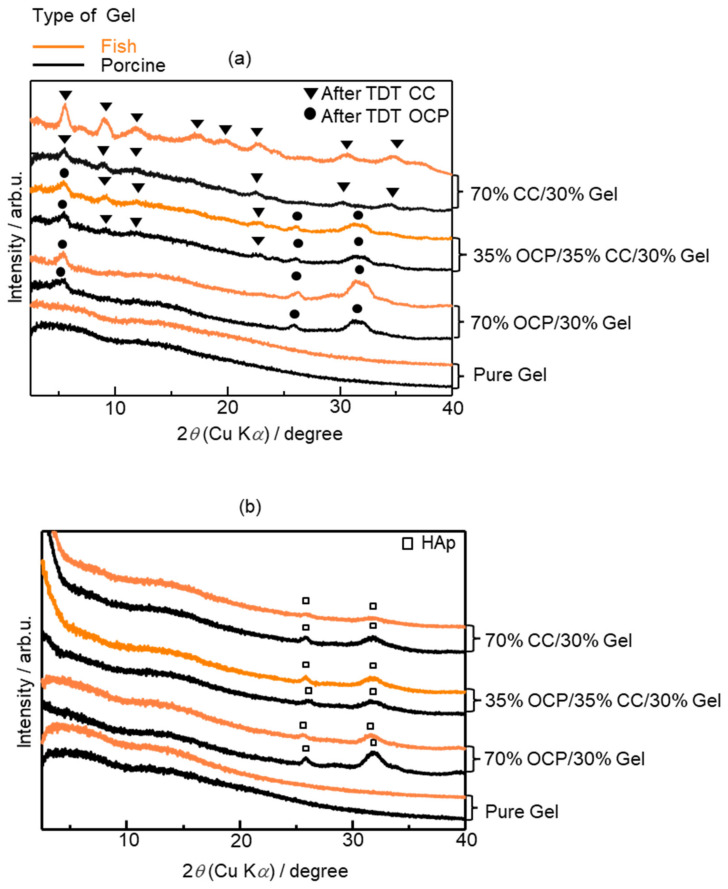
XRD patterns of samples produced using porcine and fish Gel (**a**) before SBF testing, showing initial crystalline structures of samples, and (**b**) after SBF testing, illustrating changes in crystalline structures resulting from SBF immersion.

Figure 7 shows the crystalline phases of the different Gel composites in the XRD patterns. Before immersion in the SBF, there was no significant difference between the samples containing porcine and fish Gels (Figure 7a). Following immersion in the SBF, both the samples containing porcine and fish Gels formed HAp, but the peak intensity of HAp in the porcine sample was slightly higher at the 2*θ* position of 32° compared with that of the fish Gel sample (Figure 7b).

**Figure 8:**
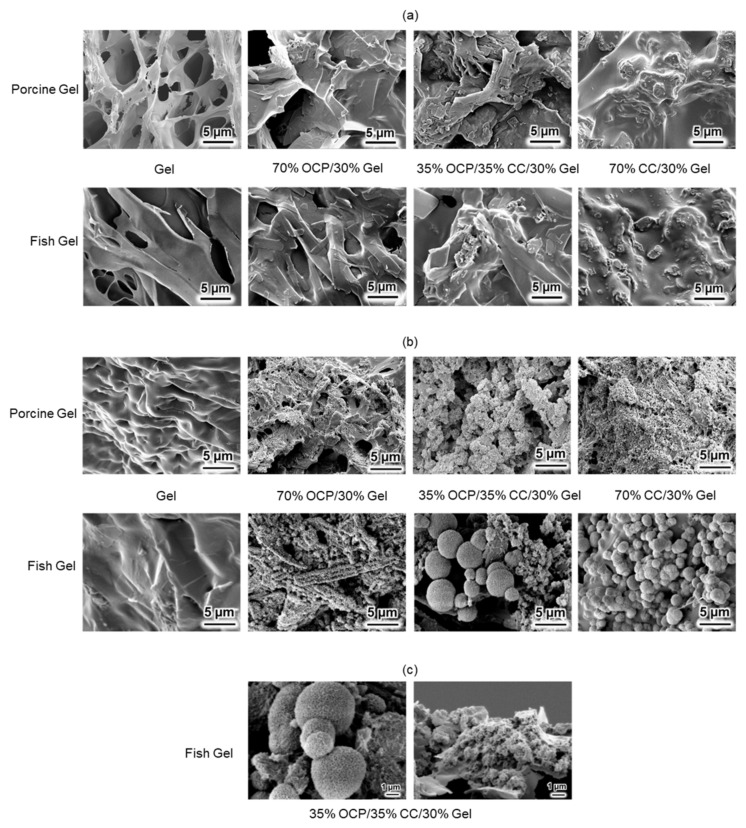
SEM images of samples produced from porcine and fish Gel (**a**) before and (**b**) after SBF testing, and (**c**) surface (left) and cross-sectional (right) views of spherical hydroxyapatite (HAp) produced from a 35% OCP/35% CC/30% fish Gel composite, illustrating morphology and internal structure of HAp spheres.

SEM was employed to observe microstructural alterations in the samples combined with either porcine or fish Gel before and after immersion in an SBF. As illustrated in Figure 8a, before immersion in the SBF, pure porcine Gel exhibited a denser structure than that of pure fish Gel, which appeared to be more relaxed. No significant differences were observed between the samples treated with either type of Gel before SBF immersion. However, post-immersion analyses revealed that the samples combined with porcine Gel formed denser and smaller HAp structures than their fish Gel counterparts did. Notably, the HAp structures in the samples prepared with fish Gel were spherical, which was distinctly different from the HAp structures in the porcine Gel samples (Figure 8b).

Further observations from Figure 8b regarding the volumetric size of the spherical HAp structures indicated that those formed in the fish Gel composites were larger in volume than those in the porcine Gel samples. This indicated a greater extent of HAp crystal growth in the fish Gel-combined samples. However, these samples did not exhibit nucleation sites as dense as those observed in the porcine Gel samples, suggesting a lower number of nucleation events.

To facilitate a more detailed observation of the spherical HAp structures, Figure 8c shows SEM images of the surface and cross-sectional views of the spherical HAp produced from 35% OCP/35% CC/30% fish Gel composites under a higher magnification. The surface imagery of the spherical HAp exhibited a porous structure. These spheres were composed of numerous smaller, interconnected particles that created an intricate network of voids and channels throughout each sphere. Furthermore, the cross-sectional view demonstrated that the formation of HAp within these spherical structures was dense and uniform throughout, instead of a mere hollow formation on the surface.

**Figure 9:**
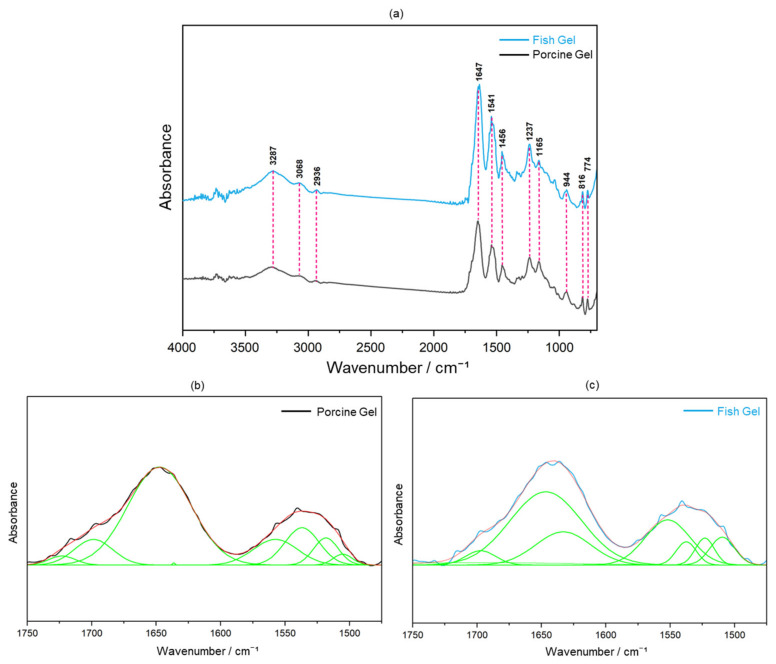
(**a**) FTIR spectra of porcine (black line) and fish Gels (blue line) after crosslinking; fitting of peaks for (**b**) porcine and (**c**) fish Gels after crosslinking in wavenumber ranges of 1750–1475 cm^−1^. The original spectra in (**b**) porcine and (**c**) fish Gels, represented by black and aqua blue lines, respectively, were recovered through cumulative peak fitting (red line), with the individual peaks displayed underneath (green lines).

FTIR spectroscopy was employed to compare the structural attributes of porcine and fish Gels after crosslinking, as shown in Figure 9a. In the spectral analysis, the four regions of 3600–2300 (Amide A), 1656–1644 (Amide I), 1560–1335 (Amide II), and 1240–670 cm^−1^ (Amide III) [21] were of interest because they were indicative of the different molecular interactions responsible for the infrared absorption. The Amide A band at 2936–3287 cm^−1^ was indicative of N–H stretching vibrations coupled with hydrogen bonding. The Amide I region at 1647 cm^−1^ corresponded to C=O stretching, influenced by hydrogen bonding and COO^−^ interactions. Amide II vibrations at 1541 and 1456 cm^−1^ were related to the N–H bond and CH_2_ deformations, respectively. The Amide III band at 1237 cm^−1^ (N–H bending) and specific stretches at 1165 (C–O stretching) and 944–774 cm^−1^ (skeletal stretching) collectively facilitated a nuanced understanding of the molecular interactions involved in infrared absorption, providing a better understanding of the molecular structure of Gels after crosslinking [22].

To further explore the nucleating effects of two different types of Gel after crosslinking on HAp, Figure 9b,c shows the spectra of the porcine and fish Gels after crosslinking, respectively, within the wavenumber range of 1750–1475 cm^−1^. The carboxy group effectively induced the nucleation of HAp in the body [23]. Due to the positive influence of carboxy groups (COO^−^) on the nucleation of HAp and the direct correlation of the Amide I region with C=O stretching vibrations, which were significantly affected by hydrogen bonding and COO^−^ interactions, the area percentage of the carboxy peak at 1647 cm^−1^ was used to indicate the content of carboxy groups in the two types of Gels. The peak area percentage of the carboxy peak relative to the peaks in the wavenumber range of 1750–1475 cm^−1^ was 57.7% and 44.9% for the porcine and fish Gels, respectively (Figure 9b,c), after crosslinking. These results indicated that the peak area percentage of the carboxy peak in porcine Gel exceeded that in fish Gel after crosslinking, suggesting a higher presence of carboxy groups on the surface of porcine Gel under crosslinking conditions, thereby implying a superior nucleation capability for HAp compared with that of the fish Gel.

## 3. Discussion

**Figure 10:**
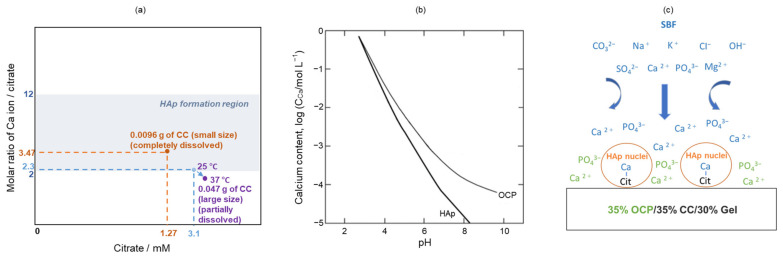
(**a**) Molar ratio of calcium to citrate and corresponding concentration of citrate required for HAp formation; orange points represent complete dissolution of 0.0094 g of CC; light blue points indicate values calculated based on solubility of 0.047 g of CC in water at 25 °C; purple points illustrate trend of movement caused by increased solubility of CC at 37 °C; (**b**) solubility curve of HAp and OCP [24]; (**c**) HAp formation of 35% OCP/35% CC/30% Gel (96-well plate) sample in SBF. Ions in blue and green generated from SBF and OCP, respectively.

As shown in Figure 5a, the shifts in the characteristic peaks towards the right for 70% OCP/30% Gel, as well as the crystal phase transformation in 70% CC/30% Gel, following the crosslinking process are evident. These changes are attributed to the high-temperature vacuum conditions applied during TDT, which likely caused dehydration in the hydrated layers of OCP and phase transformation in CC. Specifically, the thermal dehydration of OCP resulted in the narrowing of hydrated layers, leading to the observed peak shift [24]. In Figure 1, Figure 2, Figure 3, Figure 4, Figure 5 and Figure 6, the experimental results demonstrate that 0.0094 g of CC and its corresponding quantity in a 96-well plate sample could generate HAp and even synergise with OCP to promote HAp formation following SBF soaking. Conversely, 0.047 g of CC and its corresponding quantity in a 24-well plate sample not only lacked the ability to generate HAp after soaking in an SBF but also inhibited the formation of HAp under OCP conditions. The nucleation behaviour of HAp was elucidated using the relative molar ratio of calcium to citrate [18]. Figure 10a displays the HAp formation region, showing that when the molar ratio of calcium to citrate is between 2 and 12, the citrate concentration can induce HAp formation in an SBF. This is because, within this concentration range, citrate can partially chelate calcium ions to form a positively charged complex, which can adsorb negatively charged phosphate ions and other citrate ions on the calcium-citrate complex, acting as a critical size cluster that serves as a nucleus for the crystal growth of HAp. Following nucleation, HAp crystals grew spontaneously given that the SBF was already supersaturated with HAp. Given the unknown solubility of CC in an SBF at 37 °C, we used its solubility in water at 25 °C (0.95 g/L) for the calculation. The maximum solubility of CC in 30 mL of the SBF solution based on its known solubility in water is 0.0285 g. Therefore, 0.0094 g of CC can completely dissolve in 30 mL of an SBF, as it is well below the solubility limit of 0.0285 g. Moreover, as shown in Figure 1, the sample containing 0.0094 g of CC was completely converted to HAp after SBF treatment, suggesting that this sample was fully dissolved in the SBF. The calculated molar ratio of calcium to citrate and citrate concentration in 30 mL of an SBF solution were 3.47 and 1.27 mM, respectively, placing them within the HAp formation region in Figure 10a. In Figure 2, after immersion in the SBF, the sample containing 0.0094 g of CC powder dissolved into calcium and citrate ions. Citrate partially chelated calcium ions, forming critical size clusters that provided nucleation sites for HAp. Following nucleation, HAp crystals grew, as confirmed by the XRD patterns that showed the morphology transformed into rod-like clusters of HAp (Figure 2b). Furthermore, Figure 5a shows a 35% OCP/35% CC/30% Gel sample that was fully converted to HAp after undergoing SBF treatment, indicating that OCP dissolved in the SBF because of the consumption of calcium ions by the formation of calcium–citrate complexes. Figure 10b shows the solubility curves of OCP and HAp [25]. As shown in Figure 10b, when the concentration of free calcium ions in the SBF decreased to a range in which the SBF was not supersaturated for OCP but remained supersaturated for HAp, OCP began to dissolve, releasing more calcium and phosphate ions to promote HAp formation post-nucleation (Figure 10c). In the case of 35% OCP/35% CC/30% pig Gel, not only does the chelation of citrate with calcium reduce the concentration of calcium ions in the SBF, but the crosslinked pig Gel, which contains more carboxyl groups, also provides more nucleation sites for HAp than 35% OCP/35% CC/30% fish Gel. This initially consumes more calcium ions from the SBF, thereby lowering their concentration and further promoting the dissolution of OCP. This dissolution releases additional calcium and phosphate ions, which further promote HAp nucleation. This explains the synergised HAp formation exhibited by the 35% OCP/35% CC/30% pig Gel composite made using a 96-well plate, leading to the highest material surface generation rate for HAp formation observed in the SEM images (Figure 6a and Figure 8b).

When the citric acid concentration increased and the ratio of calcium to citrate decreased to below 2, the two citrate molecules formed a stable complex with the calcium ions in the form of [Ca(cit)_2_]^4−^. Consequently, a cluster larger than a critical size can hardly form, thereby inhibiting HAp nucleation. As shown in Figure 1, the sample containing 0.047 g of CC retained its structure after SBF treatment, indicating that it did not completely dissolve in the SBF. Given the unknown solubility of CC in an SBF at 37 °C, its solubility in water at 25 °C was assumed for calculating the molar ratio of calcium to citrate and citrate concentration in 30 mL of an SBF at 37 °C, which were determined to be 2.3 and 3.1 mM, respectively (Figure 10a). However, the actual temperature for SBF dissolution was 37 °C, implying that the actual solubility and citrate concentration were higher than assumed. Based on a calcium-to-citrate ion ratio of 1.5 for Ca_3_(C_6_H_5_O_7_)_2_, an increase in the solubility would reduce the molar ratio of calcium to citrate from 2.3. Therefore, the actual citrate concentration of 0.047 g of CC in 30 mL of the SBF at 37 °C would be higher than calculated, and the molar ratio of calcium to citrate would be lower. Consequently, the corresponding point in Figure 10a would move downwards and to the right (to the purple point) and fall outside of the HAp formation region, thereby preventing the formation of HAp.

As shown in Figure 8b, the samples containing porcine Gel composites exhibit superior nucleation abilities compared with those containing fish Gel composites after immersion in an SBF. However, their crystalline growth capabilities were low. This was attributed to the high nucleation capability in the initial phase, which consumed a significant amount of calcium and phosphate ions from the SBF for nucleation, thus reducing the availability of calcium and phosphate ions for crystal growth. Conversely, the fish sample group had fewer nucleation sites, leading to a lower consumption of calcium and phosphate ions for initial nucleation. This allowed for an ample amount of calcium and phosphate ions to be available for crystal growth in the later stages, resulting in the formation of spherical HAp.

## 4. Materials and Methods

### 4.1. Sample Preparation

#### 4.1.1. Synthesis of Octacalcium Phosphate

For the synthesis of OCP, 100 mL of ultrapure water was initially stirred at 60 °C and 500 rpm using a magnetic stirrer. Subsequently, 6.0 mmol of phosphoric acid (H_3_PO_4_, 85% aqueous solution, FUJIFILM Wako Pure Chemical Corporation, Osaka, Japan) and 8.0 mmol of calcium carbonate (CaCO_3_, calcite: 99.5%, Nacalai Tesque, Kyoto, Japan), respectively, were added to the water, followed by the continuous stirring of this mixture for 3 h. The pH was adjusted to 5.0 using hydrochloric acid (HCl, 1 M solution; FUJIFILM Wako Pure Chemical Corporation, Osaka, Japan), followed by 30 min of additional stirring. The final step involved vacuum filtration to collect the precipitate, which was rinsed with ultrapure water and ethanol and dried at 40 °C for at least 12 h.

#### 4.1.2. Synthesis of Octacalcium Phosphate/Calcium Citrate/Gelatine

A CC powder ([O_2_CCH_2_C(OH)(CO_2_)CH_2_CO_2_]_2_Ca_3_·4H_2_O, 99%, Sigma-Aldrich, Tokyo, Japan) was mixed with an OCP powder in different mass ratios, namely, 10:0, 5:5, and 0:10, to obtain an OCP/CC ceramic mechanical mixture. Gels derived from porcine (Food Grade; Type A; 225 Bloom, MP Biomedicals, LLC 29,525 Fountain Parkway Solon, OH 44139, USA) and fish skins (Lot No: 181128, CAS RN^®^: 9000-70-8, FUJIFILM Wako Pure Chemical Corporation, Osaka, Japan) were mixed with pure water at 75 °C to obtain a Gel concentration of 3%. Different mass ratios of the OCP and CC powders were added to the porcine and fish Gels to ensure that the mass ratio of the OCP and CC powders to Gel particles was maintained at 7:3. This resulted in slurries with compositions of 70% OCP/30% Gel, 35% OCP/35% CC/30% Gel, and 70% CC/30% Gel. Afterward, the slurries were transferred to 96- and 24-well plates (150 and 750 μL per well, respectively) and immediately frozen using liquid nitrogen to ensure a homogenised phase. Afterwards, the frozen samples were lyophilised for 48 h. For comparison, Gel samples without OCP/CC powder, which served as the control group, were prepared using the same method employed for the composite materials. Subsequently, thermal crosslinking of both the OCP/CC/Gel composites and Gel alone was conducted in a vacuum environment at a temperature of 150 °C for 24 h. The samples prepared using the 96-well plate had a thickness and diameter of 5 mm, whereas those prepared using the 24-well plate had a thickness and diameter of 5 and 14 mm, respectively.

### 4.2. Soaking in Simulated Body Fluid

The apatite-forming properties of the samples were assessed in vitro using an SBF [26]. To prepare the SBF, 800 mL of ultrapure water was introduced into a 1 L glass beaker and stirred using a magnetic stirrer. This was achieved by the step-by-step dissolution of the reagents (orders 1–9), as shown in Table 1, while ensuring that each reagent was fully dissolved before the addition of the subsequent reagent. The pH of the solution was adjusted to 7.4 at 37.0 °C using hydrochloric acid. Following this procedure, the solution was transferred to a volumetric flask, where ultrapure water was added to bring the total volume of the solution to 1 L.

Then, 0.0094 and 0.047 g of the CC powders (which corresponded to 70% CC/30%Gel for each well in the 96- and 24-well plates, respectively), 70% OCP/30% Gel, 35% OCP/35% CC/30% Gel, 70% CC/30% Gel, and Gel alone synthesised in 96- and 24-well plates were immersed in 30 mL of the fresh SBF in separate plastic tubes. To fully immerse the materials in an SBF and make them sink to the bottom of the plastic tubes, Pt wires were inserted into the materials, and the temperature of the plastic tubes was maintained at 37.0 °C for 7 d. The immersed powders and composites were collected after 7 d, washed with ultrapure water and 99% ethanol, and then dried in a drying oven at 40 °C.

### 4.3. Characterisation

The crystalline phases of the samples were characterised using a Rigaku MiniFlex 600 powder XRD (Rigaku, Tokyo, Japan), which utilised Cu *Kα* radiation with an X-ray wavelength of 0.154056 nm. The crystal morphologies of the samples were examined using SEM (JSM-IT200LAJ, JEOL Ltd., Tokyo, Japan). This step was preceded by coating the samples with a thin Au film to enhance the imaging quality. FTIR (FT/IR-6200, JASCO Corp., Tokyo, Japan) was used to determine the functional groups present in the samples. This analysis involved mixing the powdered samples with KBr granules (IR spectrophotometry-grade, Wako Pure Chemical Industries, Osaka, Japan) in a KBr:sample mass ratio of 300:1. The samples of porcine and fish Gels (after crosslinking) were directly analysed using an attenuated total reflectance accessory (PRO 410-M, JASCO Corp., Tokyo, Japan). ICP-OES (ICPE 9820, Shimadzu, Tokyo, Japan) was employed to measure the concentrations of calcium and phosphate ions in the SBF before and after the immersion of the samples to infer the formation of HAp by the samples in the SBF.

## 5. Conclusions

Small amounts of CC can synergise with OCP/Gel to enhance HAp formation in an SBF. In contrast, higher amounts of CC inhibit HAp formation with OCP/Gel in an SBF. In addition, composites containing fish Gel exhibited reduced nucleation but greater crystal growth than those containing porcine Gel. These findings contribute to a deeper understanding of the effect of bioceramic composites on HAp formation in an SBF and pave the way for the development of biomaterials for bone regeneration.

## Figures and Tables

**Table 1:** Reagents used to prepare 1 L of simulated body fluid.

Order	Reagent	Amount
1	NaCl	7.996 g
2	NaHCO_3_	0.350 g
3	KCl	0.224 g
4	K_2_HPO_4_·3H_2_O	0.228 g
5	MgCl_2_·6H_2_O	0.305 g
6	1.0 mol/L HCl	40 mL
7	CaCl_2_	0.278 g
8	Na_2_SO_4_	0.071 g
9	(CH_2_OH)_3_CNH_2_	6.057 g

## Data Availability

Data are contained within the article.
